# Supplementary material for: Cytoneme-mediated intercellular signaling in keratinocytes is essential for epidermal remodeling in zebrafish
Source: eLife. 2025 Aug 6;13:RP97400. doi: 10.7554/eLife.97400 (PMC12327944; doi:10.7554/eLife.97400)
Supplement: Figure 4—figure supplement 1—source data 1. [file elife-97400-fig4-figsupp1-data1.zip › Figure 4_Figure supplement1_Source data 1/Figure 4_Figure supplement1_Source data 1.pdf]

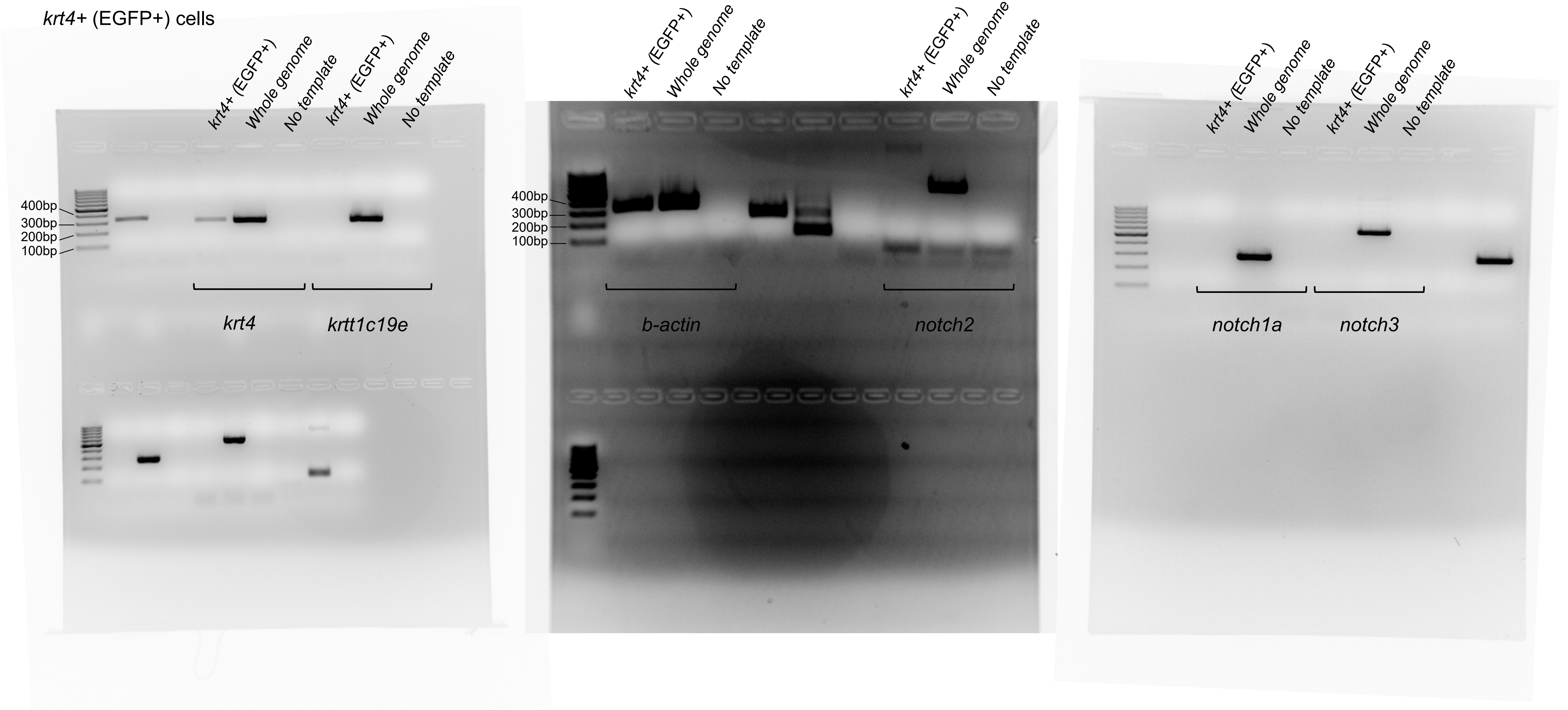

Figure 4, Figure supplement 1, Source Data 1.1 Original gels corresponding to Figure supplement 1 (Left panel). All samples were amplified using corresponding primers to detect the endogenous expression of *krt4, krtt1c19e, b-actin, notch 2, notch 1a* and *notch 3* in EGFP+ FACS-sorted cells from *Tg(krt4:lyn-EGFP)*. Samples were loaded on the gels in the following order: target, positive control (whole genome) and negative control (no template). 100bp DNA ladder was used for all gels and all molecular weight markers were employed.

krt4+ (EGFP+) cells

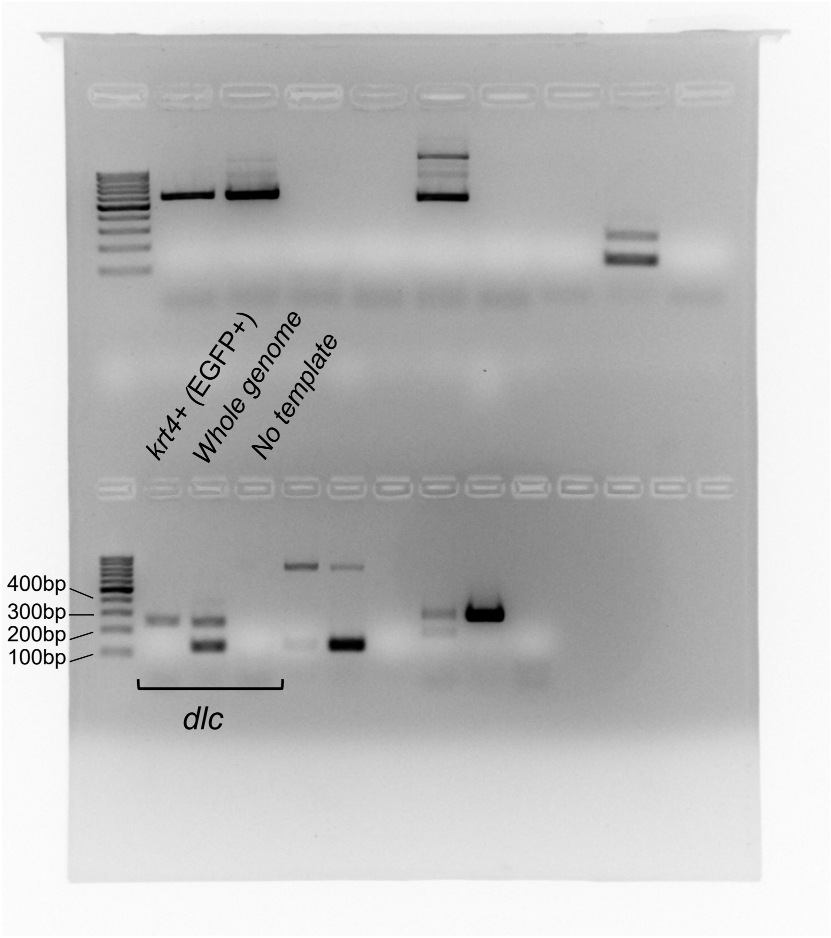

Figure 4, Figure supplement 1, Source Data 1.2 Original gels corresponding to Figure supplement 1 (Left panel). Samples were amplified using corresponding primers to detect the endogenous expression of *dlc* in EGFP+ FACS-sorted cells from *Tg(krt4:lyn-EGFP)*. Samples were loaded on the gel in the following order: target, positive control (whole genome) and negative control (no template). 100bp DNA ladder was used for all gels and all molecular weight markers were employed.

krtt1c19e+ (tdTomato+) cells

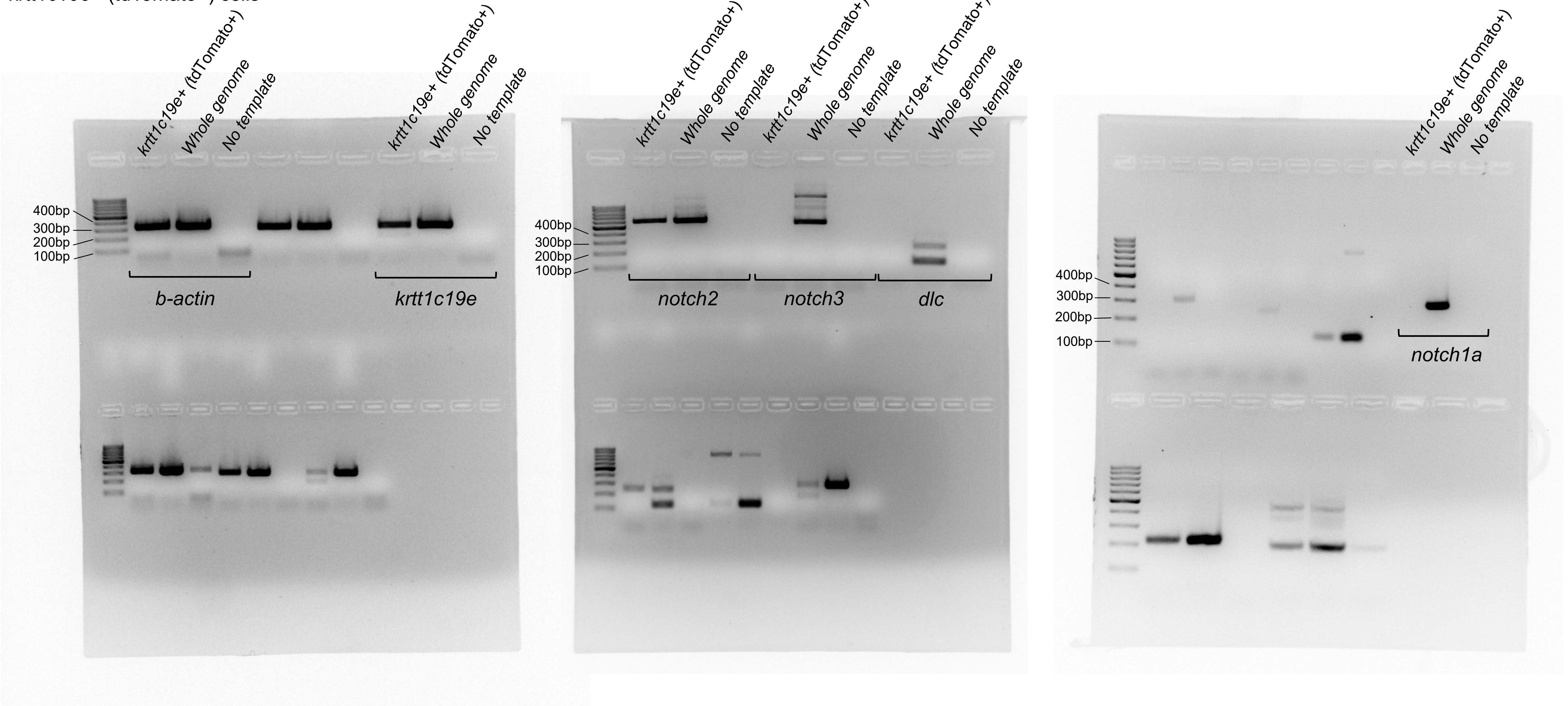

Figure 4, Figure supplement 1, Source Data 1.3 Original gels corresponding to Figure supplement 1 (Right panel). All samples were amplified using corresponding primers to detect the endogenous expression of *b-actin, krtt1c19e, notch 2, notch 3, dlc* and *notch1a* in tdTomato+ FACS-sorted cells from *Tg(krtt1c19e:tdTomato)*. Samples were loaded on the gels in the following order: target, positive control (whole genome) and negative control (no template). 100bp DNA ladder was used for all gels and all molecular weight markers were employed.

krtt1c19e+ (tdTomato+) cells

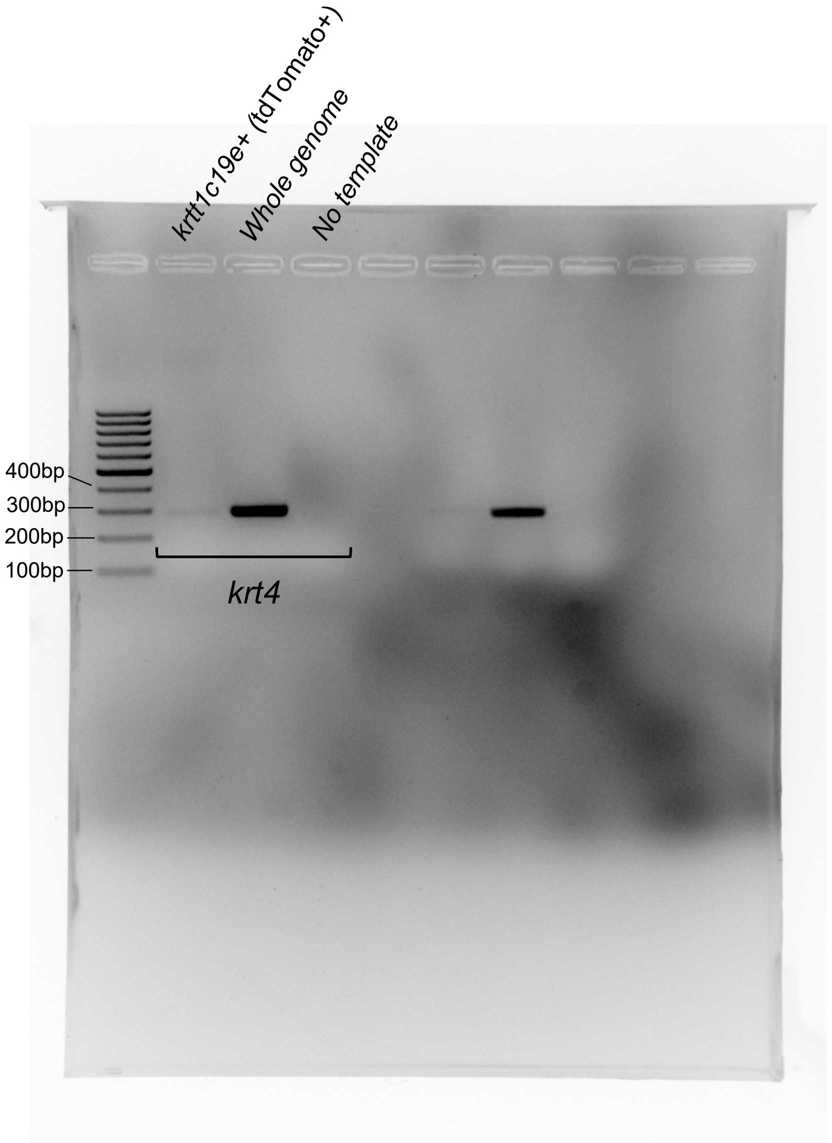

Figure 4, Figure supplement 1, Source Data 1.4 Original gels corresponding to Figure supplement 1 (Right panel). All samples were amplified using corresponding primers to detect the endogenous expression of *krt4* in tdTomato+ FACS-sorted cells from *Tg(krtt1c19e:tdTomato)*. Samples were loaded on the gel in the following order: target, positive control (whole genome) and negative control (no template). 100bp DNA ladder was used for all gels and all molecular weight markers were employed.
