# Supplementary figures and images for: Cytoneme-mediated intercellular signaling in keratinocytes is essential for epidermal remodeling in zebrafish
Source: eLife. 2025 Aug 6;13:RP97400. doi: 10.7554/eLife.97400 (PMC12327944; doi:10.7554/eLife.97400)

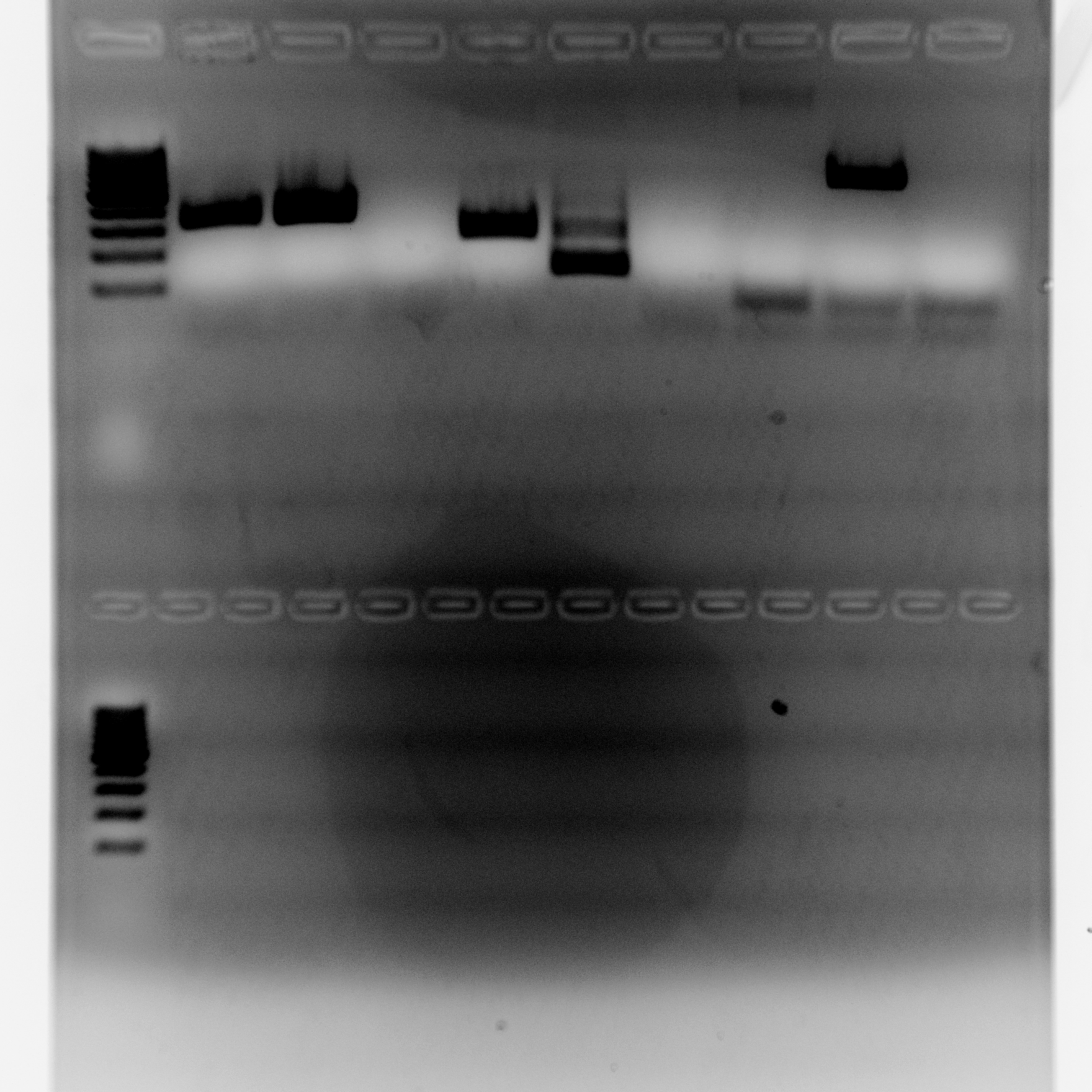

Supplement: Figure 4—figure supplement 1—source data 2. [file elife-97400-fig4-figsupp1-data2.zip › Figure 4_Figure supplement1_Source data 2/EGFP+ cells/b-actin_notch2 expression in EGFP+ cells.tif]

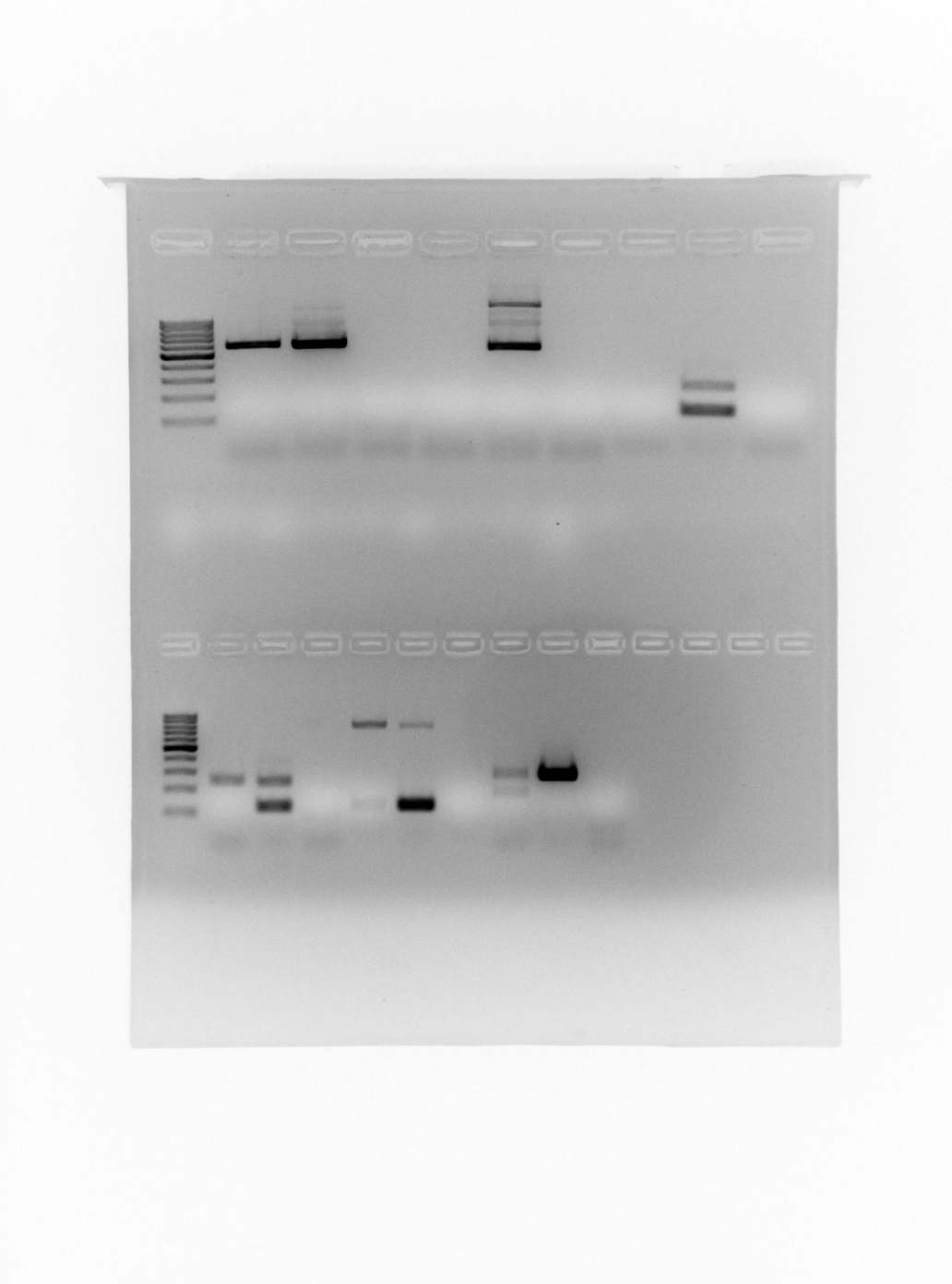

Supplement: Figure 4—figure supplement 1—source data 2. [file elife-97400-fig4-figsupp1-data2.zip › Figure 4_Figure supplement1_Source data 2/EGFP+ cells/dlc expression in EGFP+ cells.tif]

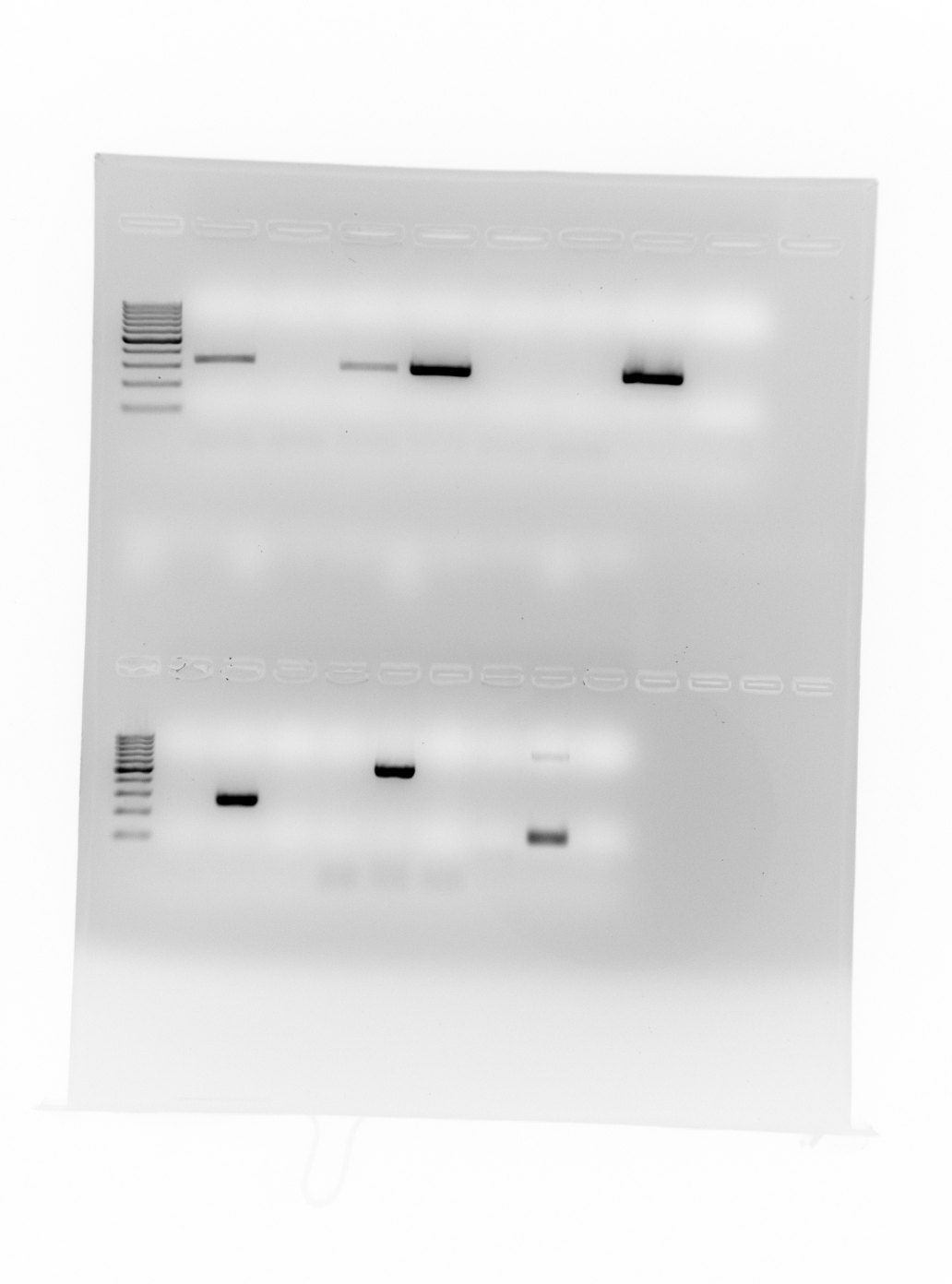

Supplement: Figure 4—figure supplement 1—source data 2. [file elife-97400-fig4-figsupp1-data2.zip › Figure 4_Figure supplement1_Source data 2/EGFP+ cells/krt4_krtt1c19e expression in EGFP+ cells.tif]

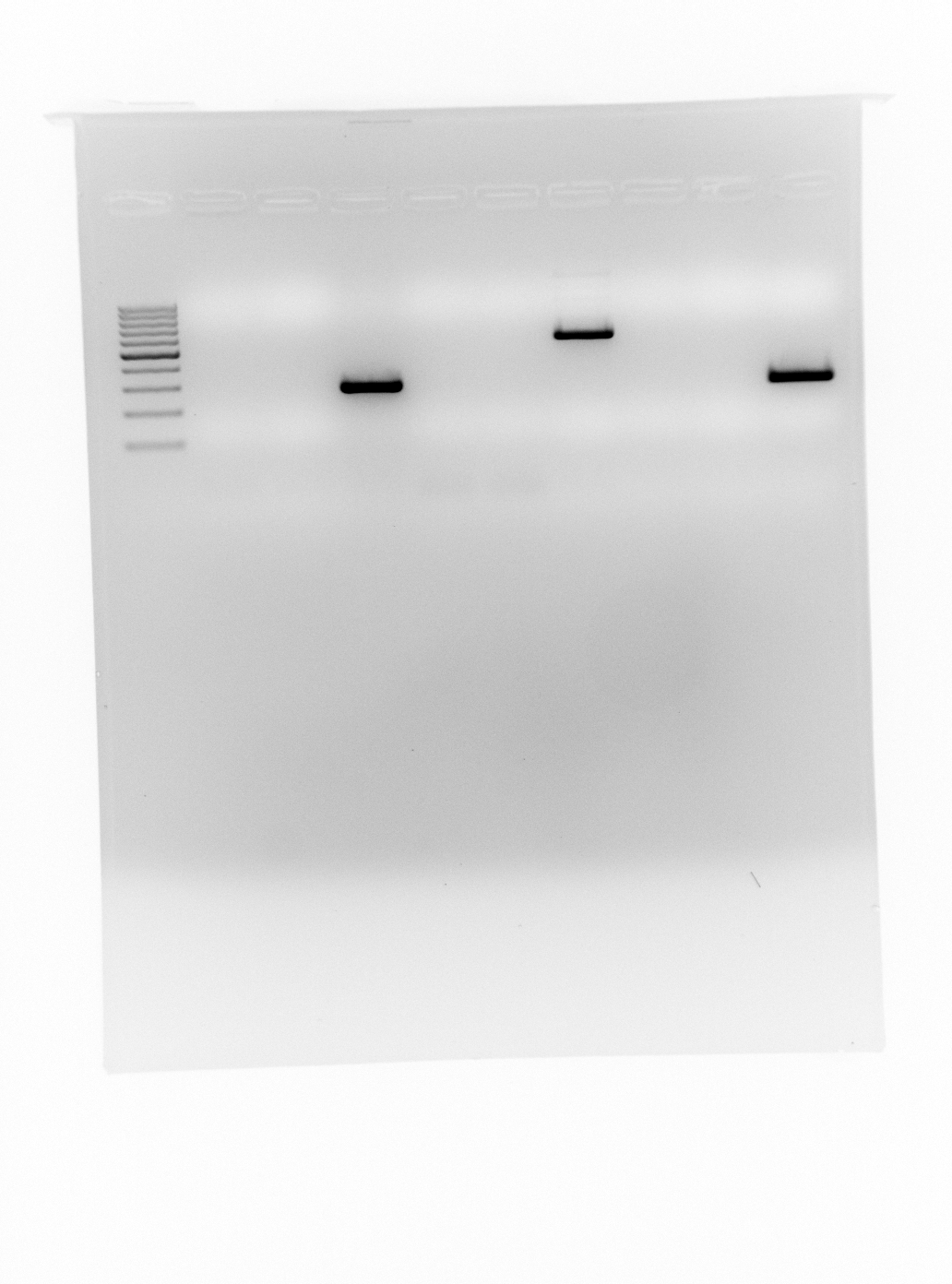

Supplement: Figure 4—figure supplement 1—source data 2. [file elife-97400-fig4-figsupp1-data2.zip › Figure 4_Figure supplement1_Source data 2/EGFP+ cells/notch1a_notch3 in EGFP+ cells.tif]

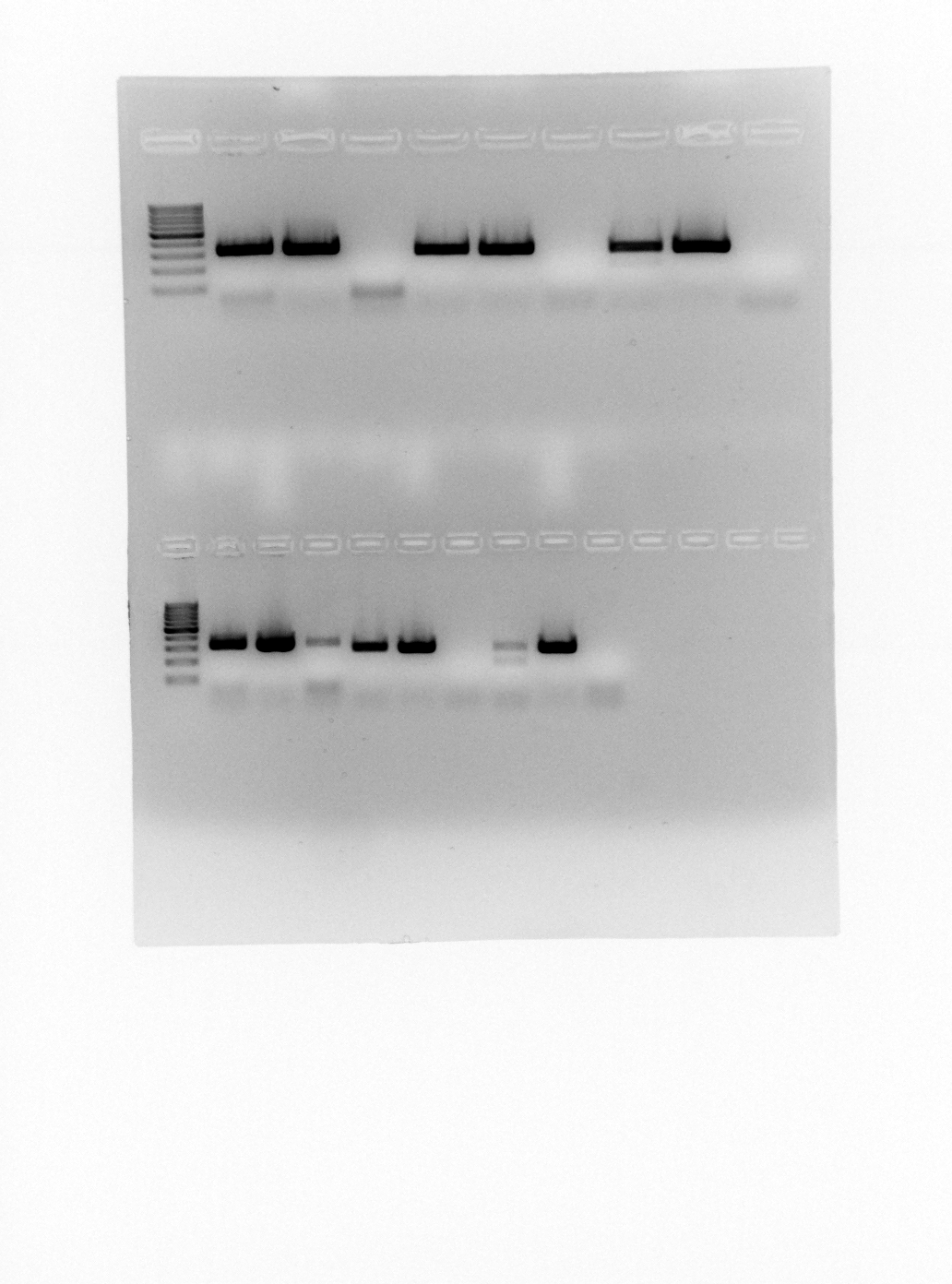

Supplement: Figure 4—figure supplement 1—source data 2. [file elife-97400-fig4-figsupp1-data2.zip › Figure 4_Figure supplement1_Source data 2/tdTomato+ cells/b-actin_krtt1c19e expression in tdTomato+ cells.tif]

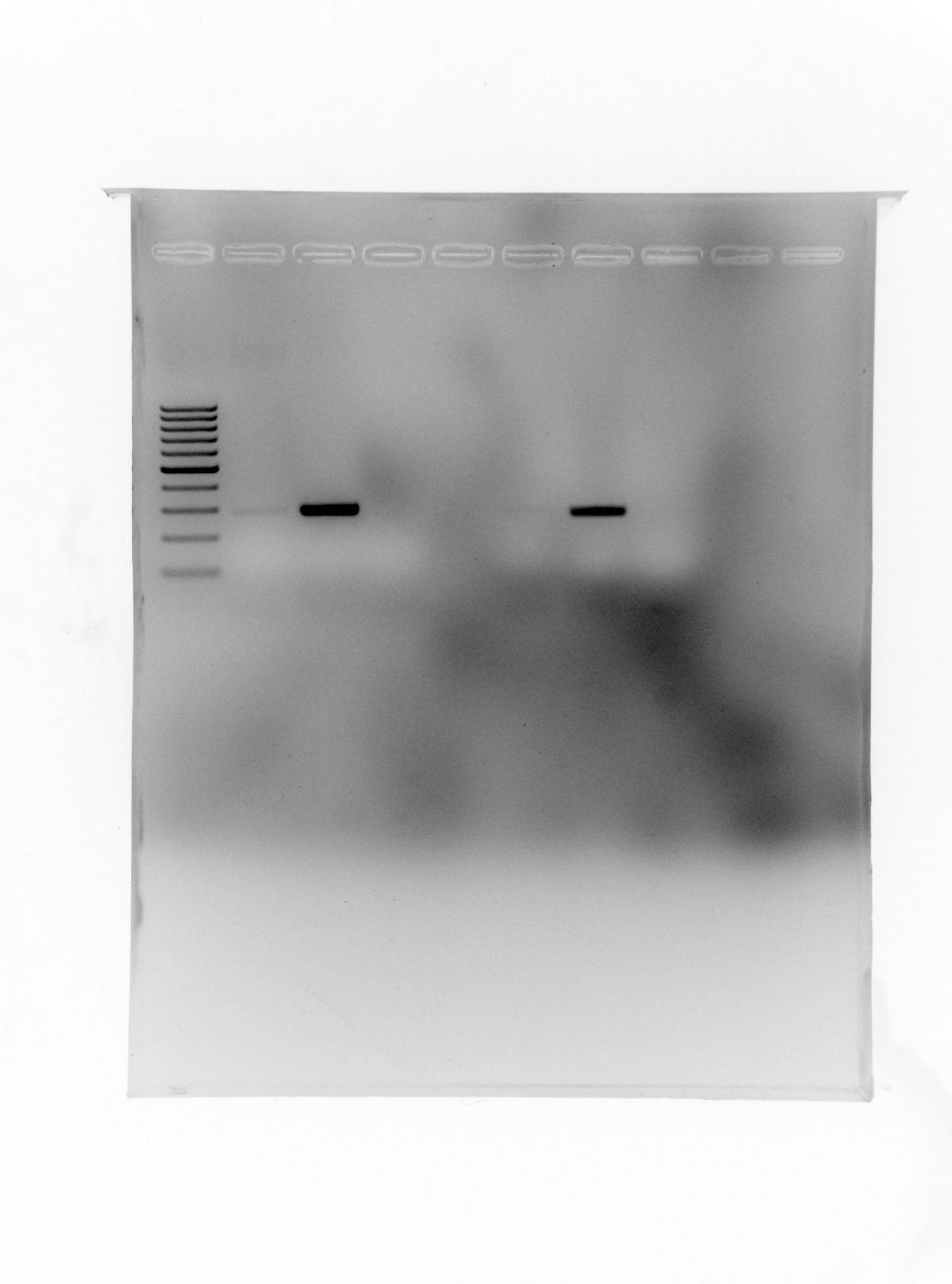

Supplement: Figure 4—figure supplement 1—source data 2. [file elife-97400-fig4-figsupp1-data2.zip › Figure 4_Figure supplement1_Source data 2/tdTomato+ cells/krt4 expression in tdTomato+ cells.tif]

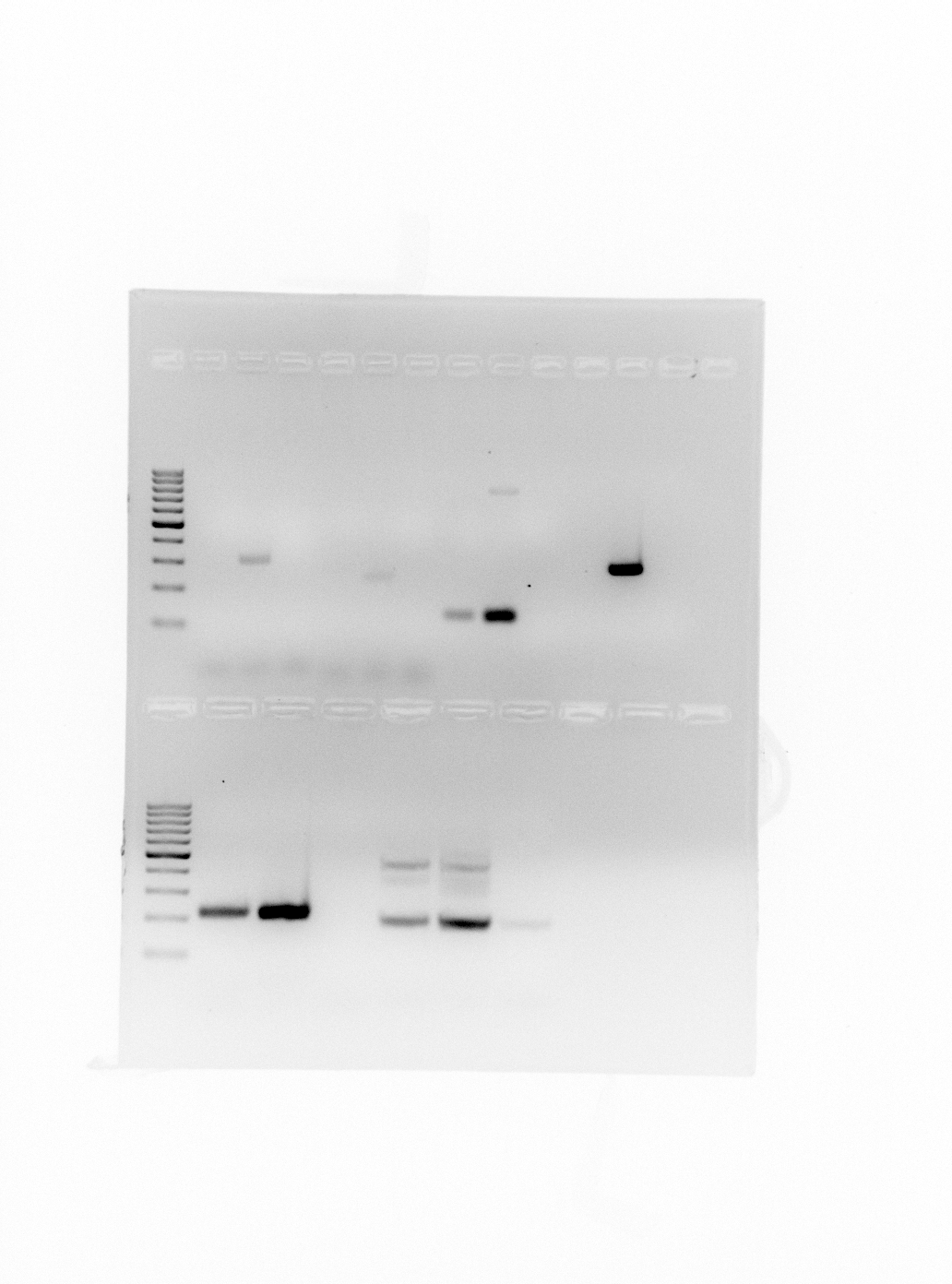

Supplement: Figure 4—figure supplement 1—source data 2. [file elife-97400-fig4-figsupp1-data2.zip › Figure 4_Figure supplement1_Source data 2/tdTomato+ cells/notch1a expression in tdTomato+ cells.tif]

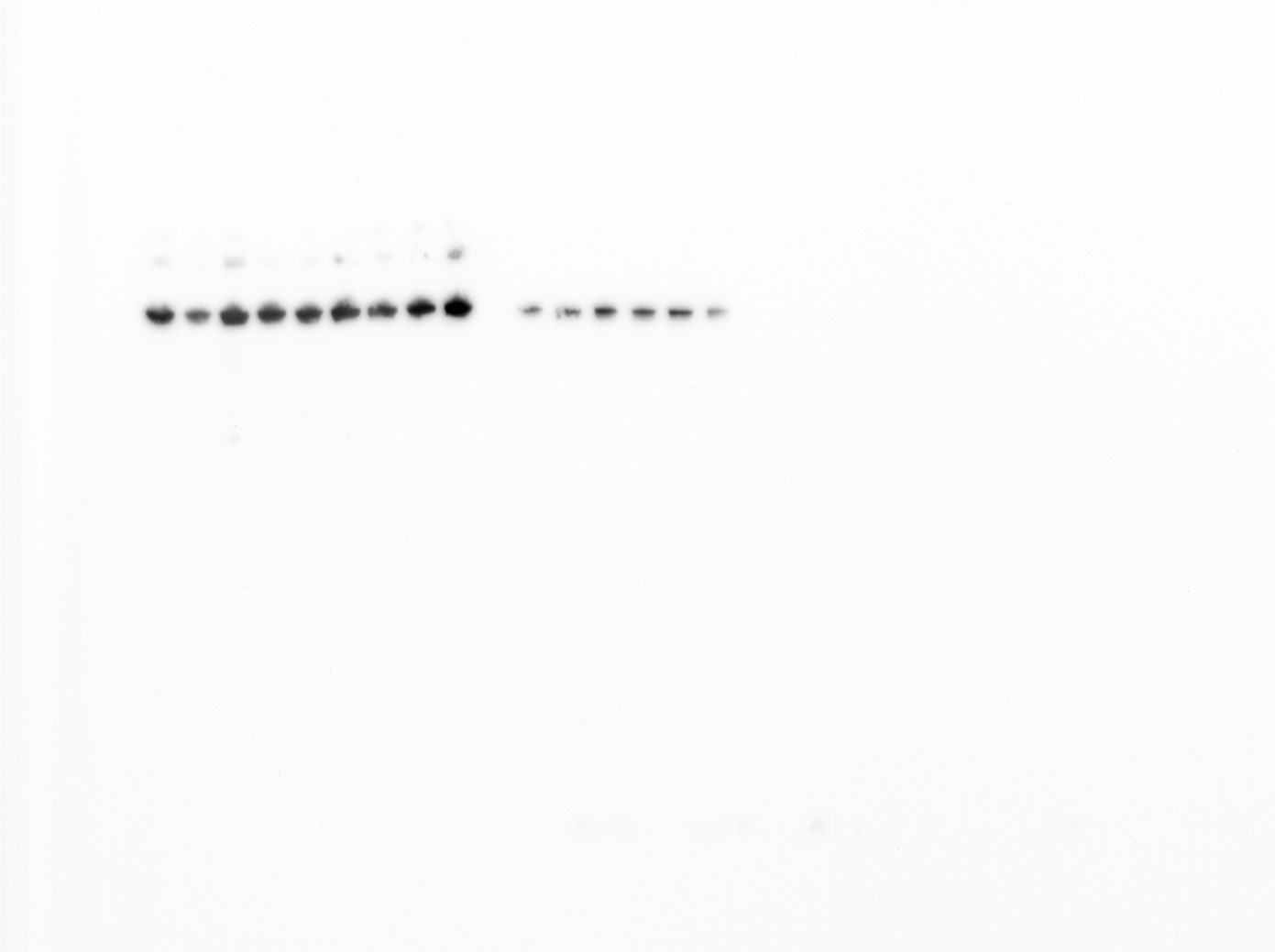

Supplement: Figure 6—figure supplement 1—source data 2. [file elife-97400-fig6-figsupp1-data2.zip › Figure 6_Figure supplement1_Source data 2/b-actin 2.tif]

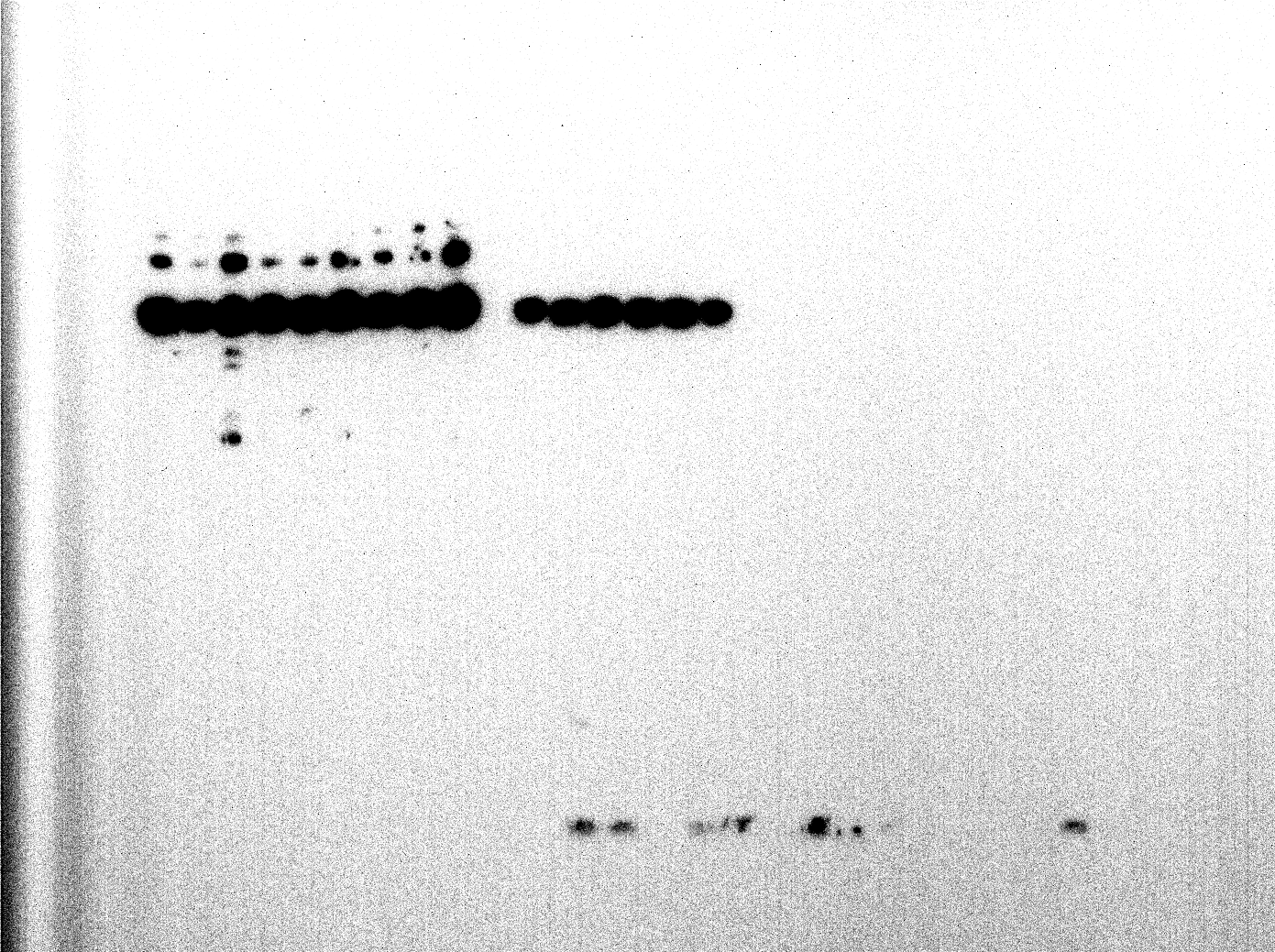

Supplement: Figure 6—figure supplement 1—source data 2. [file elife-97400-fig6-figsupp1-data2.zip › Figure 6_Figure supplement1_Source data 2/IL-17 3.tif]

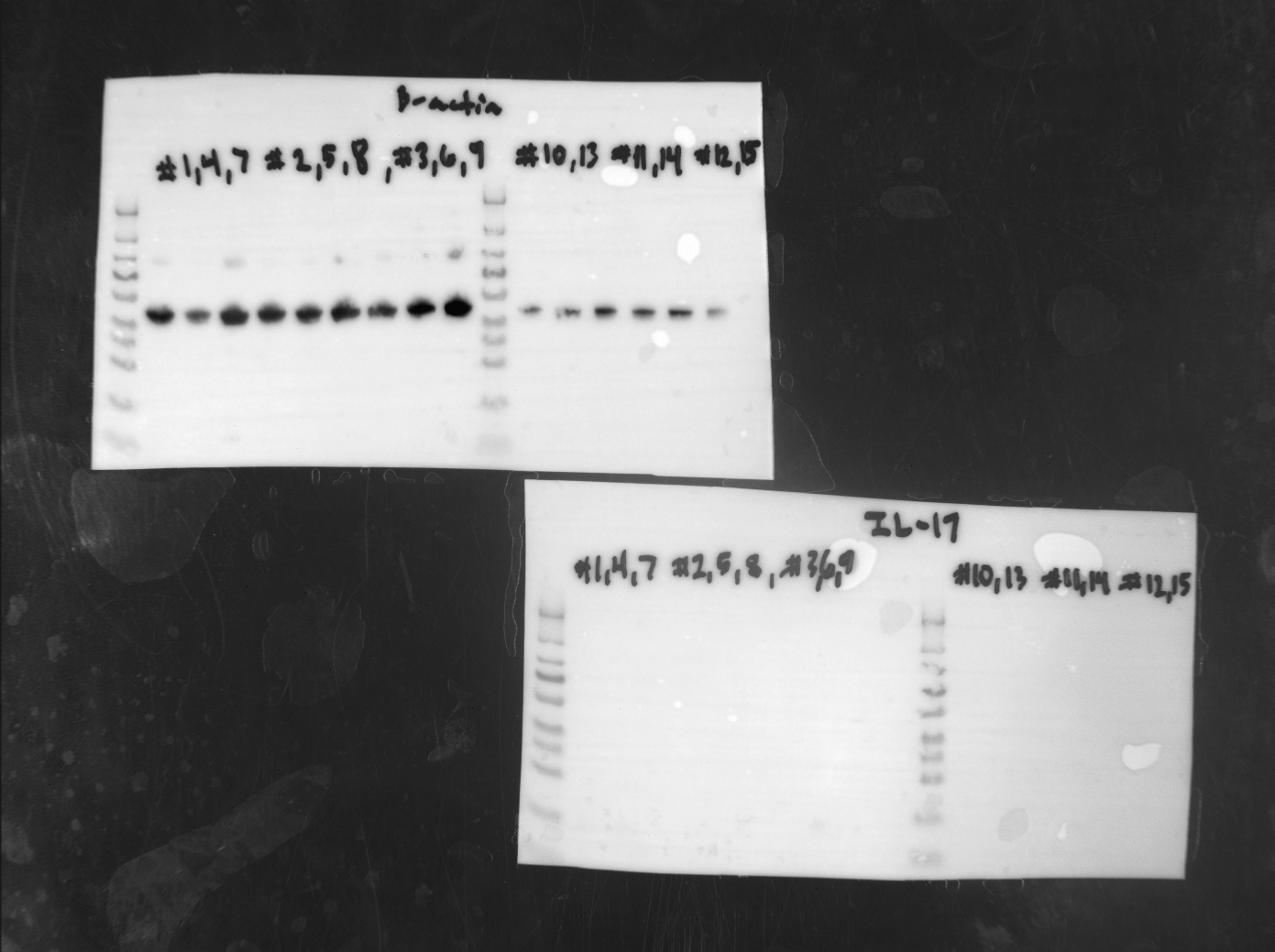

Supplement: Figure 6—figure supplement 1—source data 2. [file elife-97400-fig6-figsupp1-data2.zip › Figure 6_Figure supplement1_Source data 2/Ladder.tif]
