# Supplementary material for: Cytoneme-mediated intercellular signaling in keratinocytes is essential for epidermal remodeling in zebrafish
Source: eLife. 2025 Aug 6;13:RP97400. doi: 10.7554/eLife.97400 (PMC12327944; doi:10.7554/eLife.97400)
Supplement: Figure 6—figure supplement 1—source data 1. [file elife-97400-fig6-figsupp1-data1.zip › Figure 6_Figure supplement1_Source data 1/Figure 6_Figure supplement1_Source data 1.pdf]

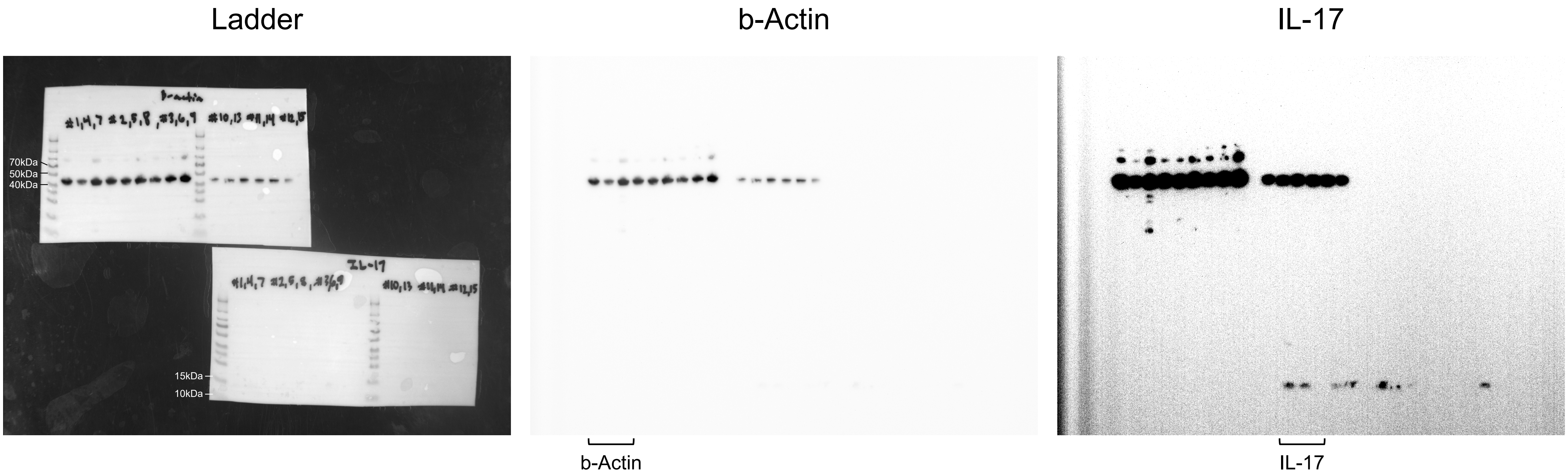

Figure 6, Figure supplement 1, Source Data 1. Original membranes corresponding to Figure supplement 1. First three lanes correspond to wild-type, heterozygous *il17a* CRISPR mutant and homozygous *il17a* CRISPR mutant. All molecular weight markers were employed.
